# Role of Chiral Configuration in the Photoinduced Interaction of D- and L-Tryptophan with Optical Isomers of Ketoprofen in Linked Systems

**DOI:** 10.3390/ijms22126198

**Published:** 2021-06-08

**Authors:** Aleksandra A. Ageeva, Ilya M. Magin, Alexander B. Doktorov, Victor F. Plyusnin, Polina S. Kuznetsova, Alexander A. Stepanov, Alexander A. Alekseev, Nikolay E. Polyakov, Tatyana V. Leshina

**Affiliations:** 1 Voevodsky Institute of Chemical Kinetics and Combustion, 630090 Novosibirsk, Russia; al.ageeva@gmail.com (A.A.A.); magin@kinetics.nsc.ru (I.M.M.); plyusnin@kinetics.nsc.ru (V.F.P.); Pol0596@yandex.ru (P.S.K.); stepanov@kinetics.nsc.ru (A.A.S.); Alexb954@mail.ru (A.A.A.); polyakov@kinetics.nsc.ru (N.E.P.); leshina@ngs.ru (T.V.L.); 2 Department of Natural Sciences, Novosibirsk State University, 630090 Novosibirsk, Russia

**Keywords:** chiral linked systems, diastereomers, enantiomers, electron transfer, resonance energy transfer, magnetic dipole–dipole interaction of electrons

## Abstract

The study of the L- and D-amino acid properties in proteins and peptides has attracted considerable attention in recent years, as the replacement of even one L-amino acid by its D-analogue due to aging of the body is resulted in a number of pathological conditions, including Alzheimer’s and Parkinson’s diseases. A recent trend is using short model systems to study the peculiarities of proteins with D-amino acids. In this report, the comparison of the excited states quenching of L- and D-tryptophan (Trp) in a model donor–acceptor dyad with (*R*)- and (*S*)-ketoprofen (KP-Trp) was carried out by photochemically induced dynamic nuclear polarization (CIDNP) and fluorescence spectroscopy. Quenching of the Trp excited states, which occurs via two mechanisms: prevailing resonance energy transfer (RET) and electron transfer (ET), indeed demonstrates some peculiarities for all three studied configurations of the dyad: (*R,S*)-, (*S,R*)-, and (*S,S*)-. Thus, the ET efficiency is identical for (*S,R*)- and (*R,S*)-enantiomers, while RET differs by 1.6 times. For (*S,S*)-, the CIDNP coefficient is almost an order of magnitude greater than for (*R,S*)- and (*S,R*)-. To understand the source of this difference, hyperpolarization of (*S,S*)-and (*R,S*)- has been calculated using theory involving the electron dipole–dipole interaction in the secular equation.

## 1. Introduction

Establishing differences in the properties of L- and D-tryptophan (Trp) which are part of various proteins, enzymes, and receptors is an important fundamental and practical problem, as the optical isomers themselves are identical in physicochemical properties, while proteins containing L- and D-isomers of amino acids, in particular, Trp, are dramatically different [1]. Thus, chiral inversion, which as is known, occurs during aging of living organisms and leads to the replacement in a number of proteins L-isomers of amino acids with D-analogs, is currently considered one of the main causes of Alzheimer’s, Parkinson’s, type II diabetes, and a number of other pathological conditions [1,2]. Due to the difficulties arising in the study of these highly disordered proteins by modern physicochemical methods, including high-resolution NMR spectroscopy, the model systems are proposed for studying the reasons of the abnormal behavior of proteins with D-optical isomers of amino acids [3]. In particular, the authors of [3] propose to apply mathematical modeling to study the role of the optical configuration of proteins by the example of short peptides. Our study puts forward a modification of this approach: the use of model systems, dyads, containing L- and D-Trp and a chiral acceptor, for studying the optical configuration influence on the photoinduced elementary processes—electron transfer (ET) and Förster resonance energy transfer (RET). As Trp is one of the few fluorescent amino acids, a set of physicochemical methods can be used in the study of the comparative reactivity of its optical isomers in elementary processes [4]. Meanwhile, the joint application of chemically induced dynamic nuclear polarization (CIDNP) and fluorescence spectroscopy techniques to study photoinduced charge transfer processes in a number of dyads, including a chiral nonsteroidal anti-inflammatory drugs (NSAIDs) and L-Trp, or another chiral donor, has indeed demonstrated the effect of optical configuration on reactivity [5,6,7,8,9,10,11,12,13,14]. This report is, to a certain extent, a development of previous study [14], in which the properties of S and R KP in (R/S) KP –(S) Trp dyad were compared. While this work is devoted to the examination of the peculiarities of the excited singlet state quenching of L- and D-Trp, being a part of dyads. These are the dyads with L-*(S-)* or D-*(R-)* have Trp residues linked with NSAID *(R)*- and *(S)*-ketoprofen (KP) (Figure S1). Degradation of the photoexcited state has been studied by NMR techniques, CIDNP and fluorescence spectroscopy. CIDNP plays a special role in this article—it is intended to highlight previously unknown differences between the optical configurations of the dyad, we will briefly describe the method [8,9]. Hyperpolarization—signals in the NMR spectrum with a non-Boltzmann population of nuclear spin sublevels, arises as a result of the interaction of electron and nuclear spins with each other and with an external magnetic field. The spin Hamiltonian describing these interactions is presented below:

(1) $$\hat{H}=g_{1}\beta B{\hat{S}}_{1z}+g_{2}\beta B{\hat{S}}_{2z}+\sum_{i}a_{1i}{\hat{S}}_{1z}{\hat{I}}_{iz}+\sum_{k}a_{2k}{\hat{S}}_{2z}{\hat{I}}_{kz}+{\hat{V}}_{DD}$$

where $g_{1}$ and $g_{2}$ are g-factors of electrons, *β* is the Bohr magneton, *B* is external magnetic field induction, ${\hat{S}}_{1z}\;$, ${\hat{S}}_{2z}\;$and ${\hat{I}}_{iz}\;,\;{\hat{I}}_{kz}$ are electron and nuclear spins operators (projection on *B* direction), and a is the HFI (hyperfine interaction) constant. The inclusion of the last term of this equation, the magnetic dipole–dipole interaction of electrons (${\hat{V}}_{DD}$), is considered for the first time in this work. ${\hat{V}}_{DD}$ is associated with the features of hyperpolarization in chiral systems. Hyperpolarization arises in the act of singlet–triplet evolution in a radical pair and manifests itself in the products of recombination and products formed in the bulk. This is the so-called $S-T_{0}\;$approximation, within which CIDNP is formed in a high magnetic field when the reaction is carried out in the probe of NMR spectrometer. As a result of the analysis of the CIDNP effects, information can be obtained on the radical stages of the process, in particular, on the distribution of the spin density in the radical precursors of the products. 

Besides, as the appearance of “hetero” structures in proteins leads to fatal consequences, quantum-chemical calculations have been used to trace the difference in conformations of “hetero” ((*R,S)-* and (*S,R)-*) and “homo” ((*S,S)-* and (*R,R)-*) configurations of the “ketoprofen-tryptophan” dyad (KP-Trp).

## 2. Results and Discussions

### 2.1. NMR and CIDNP Study

**Figure 1:**
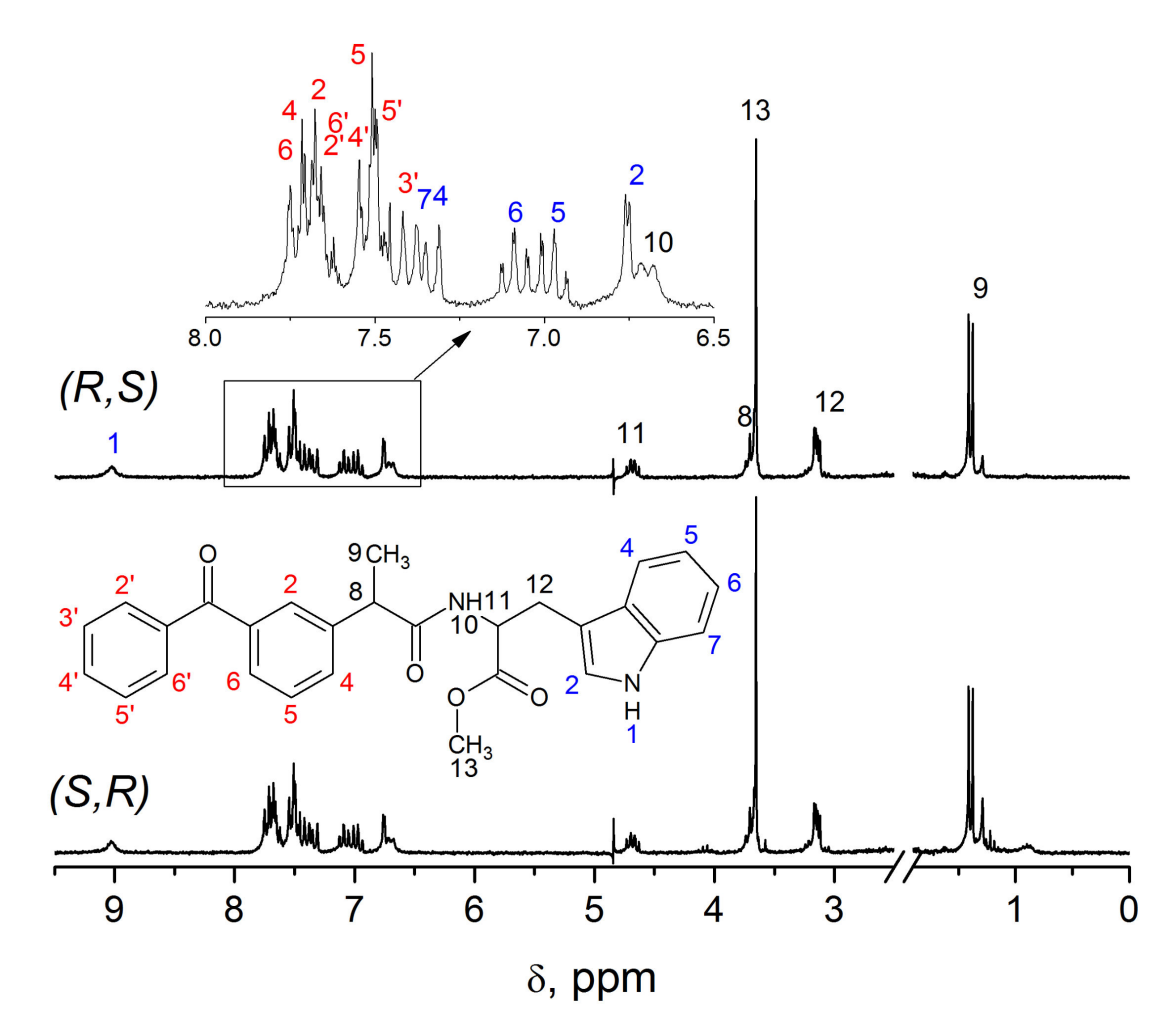
^1^H NMR spectra of 5 mM KP-Trp in acetonitrile-d_3_.

Analysis of ^1^H NMR spectra, including 2D COSY, showed the complete identity of the *(S,R)-* and *(R,S)*-enantiomers of KP-Trp dyad, as follows from the rules of stereochemistry (Figure 1 and Supplementary Figures S2–S4). 

**Figure 2:**
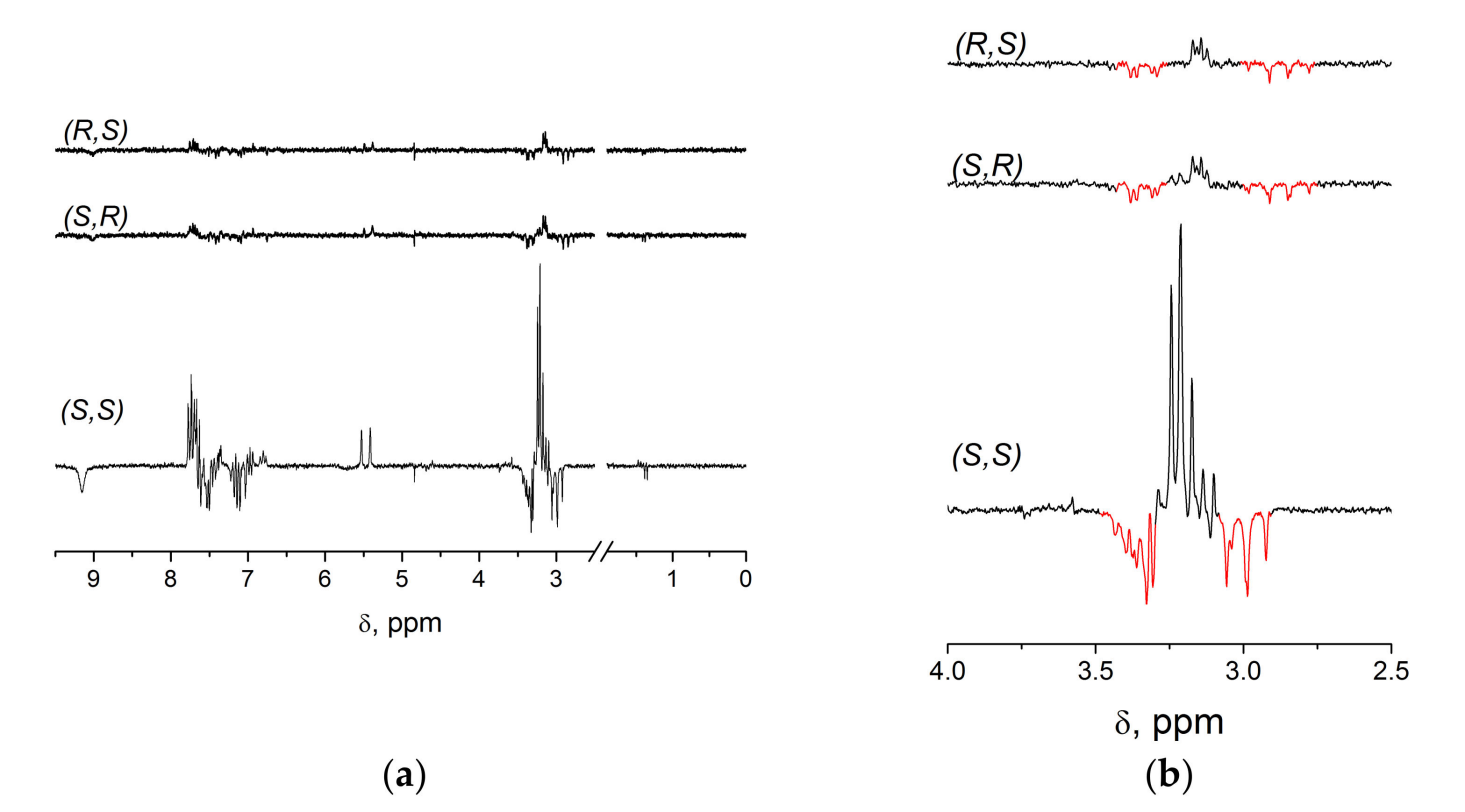
(**a**) CIDNP spectra of *(R,S)*-, *(S,R)*-, and *(S,S)*-configurations of the KP-Trp dyad in acetonitrile-d*_3_*; (**b**) the expanded region of methylene protons of the Trp fragment; red colored lines are byproducts of partial degradation of the Trp fragment of the dyad (see details in Supplementary Figures S5 and S6).

Under UV irradiation of dyad diastereomers, in accordance with previous studies for systems with *(S)*-Trp, the CIDNP effects (Figure 2) are characteristic for reversible intramolecular electron transfer from Trp in singlet excited state to KP in its ground state [5,14]. 

**Scheme 1:**
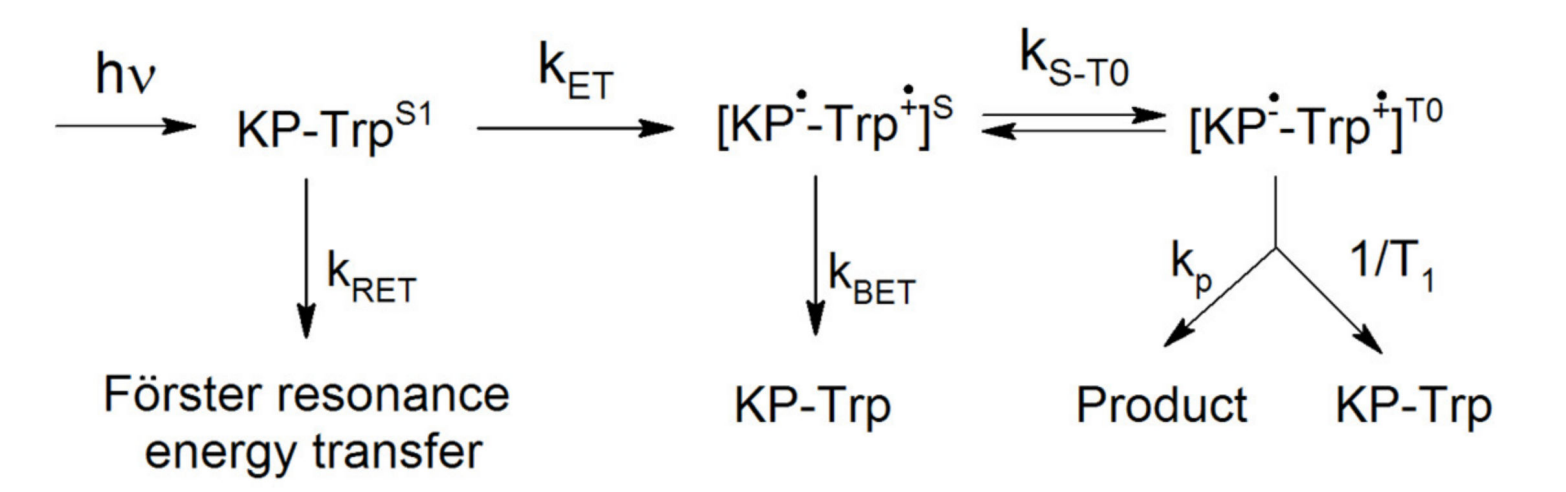
Paths of excitation degradation in donor-acceptor linked system. In this scheme, S^1^ is the singlet excited state of Trp, k_ET_ is the rate constant of electron transfer, k_RET_ is the rate constant of singlet-singlet energy transfer, S and T_0_ are the collective spin states of the biradical-zwitterion in high magnetic field, k_S-T0_ is the intersystem crossing constant, k_BET_ is the rate constant of back ET, and k_P_ is the rate constant of the byproducts formation.

Scheme 1 includes all photoinduced transformations of the KP-Trp dyad. In the part of CIDNP, there exists a special case when hyperpolarization is formed at the act of back of electron transfer in a linked system [6]. According to Scheme 1, the different CIDNP signs of initial dyad and Trp degradation byproducts (polarized multiplets located on both sides of the methylene fragment, red on Figure 2b) allow one to suppose that by-products were formed after the loss of spin correlation in triplet state of biradical-zwitterion [BZ]. Note that this situation (opposite signs of the CIDNP of the initial dyads and the products of its partial photodegradation) is also typical for other systems, in which products are formed from BZ and biradicals: “ketoprofen-aminocholestan” and “ketoprofen-N-methylpyrrolidine” dyads [12,13]. 

As can be seen from Figure 2, the CIDNP effects of (*S,R*)-configuration are similar to those for the (*R,S*)-analog, while their CIDNP coefficients remain appreciably lower than for (*S,S*)-configuration (Table 1). Observed CIDNP enhancement coefficients (K, the ratio of the intensities of the polarized signals to the equilibrium ones) of two groups of protons in dyad’s optical isomers are listed in Table 1. 

According to the authors of [8,9], from the values of the CIDNP enhancement coefficients (**K**) per one radical pair, it is possible to extract the concentrations of biradical-zwitterions (BZ) formed as a result of electron transfer in *(R,S)*- and *(S,R)*-enantiomers:

(2) $$K\;=\frac{I_{\mathrm{pol}}^{\mathrm{RS}}\times I_{\mathrm{eq}}^{\mathrm{SR}}\times {\left[\mathrm{BZ}\right]}_{\mathrm{SR}}}{I_{\mathrm{eq}}^{\mathrm{RS}}\times I_{\mathrm{pol}}^{\mathrm{SR}}\times {\left[\mathrm{BZ}\right]}_{\mathrm{RS}}},$$

where I_pol_ is the integral intensity of polarized signals in CIDNP spectrum, I_eq_ is the integral intensity of the same signal in NMR spectrum, and [BZ] is the concentration of biradical-zwitterion. Due to the equality of the CIDNP coefficients of *(R,S)*- and *(S,R)*-enantiomers, the ratio of BZs concentration is equal to 1. Because, as a first approximation, contribution of ET channel into the quenching of the Trp excited singlet state is proportional to the CIDNP efficiency in dyad’s enantiomers, it allows one to assume that this contribution is identical for the *(R,S)*- and *(S,R)*-configurations. Following this logic we must further assume that for (*S,S*)-KP-Trp contribution of ET channel is remarkably higher (approximately one order of magnitude) than in *(R,S)*- and *(S,R)*-analogs. However, note that this estimation is made without accounting the contribution to the hyperpolarization of so-called spin selectivity (K_S_) that is the difference between CIDNP coefficients of different optical configuration, described earlier in detail in [8,9]. The K_SS_ value for (*S,S)/(R,S)* ratio could be extracted from CIDNP of related systems, but the referring to such systems has led to conflicting results. Therefore, the K_S_ *(R,S)/(S,S)* value for the dyad “naproxen-tryptophan” is ~1.75 (for details see Supplementary Table S1). On the other hand, in another comparable system: the “ketoprofen-N-methylpyrrolidine” dyad, the value of K_S_ is in region of 1. We have to admit that the complexity of the processes occurring in the dyads used for comparison, does not allow us to correctly estimate the contribution from the spin selectivity to K_S_ value for *(S,S)*-configuration [9,12]. The alternative assumption that such a large spread in K_S_ values is due to the complexity of the spin selectivity itself should not be excluded from consideration. It may include, in addition to the difference in the values of the hyperfine interaction constants of various optical configurations described earlier in the works [8,9], also some other factors. An indirect confirmation of this assumption is the fact that the observed CIDNP coefficient for the *(S,S*) and the coefficients in analogous systems are of the same order of magnitude, while the coefficients for *(S,R)* and *(R,S)* are anomalously small.

### 2.2. Electron Dipole–Dipole Interaction as a Factor of the Chiral Centers’ Influence on CIDNP (Theoretical Part)

The next part is devoted to the consideration of an alternative possibility of the action of chiral centers on CIDNP. We assumed that one of these factors may be related with the influence of the electron dipole–dipole interaction on the CIDNP efficiency in different optical configurations. To elucidate the possibility of the magnetic dipole–dipole interaction influence on the stationary nuclear polarization value, solutions of the equations for the stationary density matrix elements have been performed. A description of quantum transitions between the singlet S and triplet ${Т}_{0}$ spin states under the action of spin interactions was made. This is the Zeeman interaction of the dyad’s electrons with an external strong magnetic field, HFI and magnetic dipole–dipole interaction of electrons. The Zeeman interaction of nuclei with an external magnetic field was neglected (the details see in Supplementary Materials Section S6). For simplicity we used static model of the pair neglecting of reactants mobility. A more complex model will be considered elsewhere. An analysis of the obtained analytical expressions for the stationary polarization has showed that the dipole–dipole interaction is indeed capable of changing the magnitude of hyperpolarization. The dipole–dipole magnetic interaction leads to the splitting of the spin levels S and$\;T_{0}$, resulting in the decrease of intensity of the singlet–triplet transitions caused by the difference between the Larmor frequencies and HFI. Stationary polarization decreases with increasing the dipole–dipole interaction. As the magnitude of dipole–dipole interaction, as it well known, depends on the distances and angles between nuclei, where unpaired electrons are localized, the differences in these values for (*S,S*)- and (*R,S*)-configurations of the linked system will lead to differences in the values of hyperpolarization. Using the data of quantum chemical calculations for geometry of (*S,S*)- and (*R,S*)-configurations (see Supplementary Figures S7–S13), it is possible to get the following spin selectivity contributions to CIDNP value: from K_S_ (*S,S*)/(*R,S*) ≈ 0.9 to K_S_ (*S,S*)/(*R,S*) ≈ 7 (calculation details see in Supplementary Materials). Therefore, dipole–dipole interaction really can change hyperpolarization ratio of different dyad’s optical configuration.

### 2.3. Fluorescence Measurement

**Figure 3:**
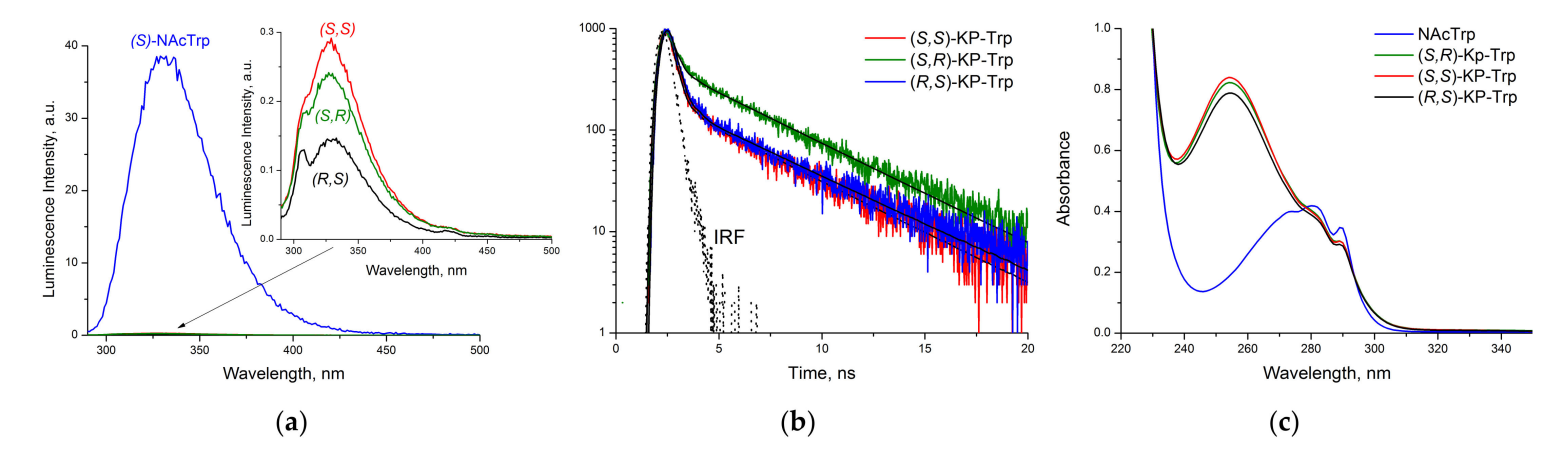
(**a**) Fluorescence spectra of is isoabsorptive solutions of *(S)*-NAcTrp and *(S,S)*-, *(S,R)*-, and *(R,S)*-optical isomers of KP-Trp dyad in acetonitrile (λ_ex_ = 280 nm) in 1 cm cuvette. Concentrations were about 6 × 10^−5^ M; (**b**) Fluorescence decay traces of (*S,S*)-, (*S,R*)-, (*R,S*)-optical isomers of KP-Trp dyad in acetonitrile at 330 nm (λ_ex_ = 270 nm), Instrument response function (IRF); (**c**) absorbance spectra of (*S*)-NAcTrp and (*S,S*)-, (*S,R*)-, and (*R,S*)-optical isomers of KP-Trp dyad in acetonitrile in 1 cm cuvette.

The emission and absorption spectra of (*R,S*)-, (*S,R*)-, and (*S,S*)-optical isomers of KP-Trp dyad in comparison with (*S*)-N-acetyltryptophan ((*S*)-NAcTrp) in acetonitrile are shown in Figure 3.

The fluorescence decay traces of the (*R,S*)- and (*S,R*)-enantiomers of KP-Trp dyad, as well as the previously studied (*S,S*)-analog [14], are described by two exponential model (see Table 2). As a rule, multiexponential fluorescence decay of Trp, as a part of protein and peptide, associated with short-lived and long-lived rotamers of Trp. There are a several models proposed to explain multi-exponential fluorescence decay [15,16,17]. 

Asfollows from the data in Table 2, there is a remarkable difference in fluorescence decay in (*S,R*)- and (*R,S*)-enantiomers: distribution of times A_1_ and A_2_ is different for (*S,R*)-enantiomer in comparison with other analogues. This seems to be the result of small contribution of short-lived rotamers.

Besides, fluorescence decay of Trp is well known to be complicated due to high sensitivity to local environment [16]. However, in our case note that time distribution weakly depends on solvent polarity. Another important characteristic is the shifts of the emission maxima of Trp in different polarities, which are associated with the predominance of the ^1^L_a_ and ^1^L_b_ transitions [17]. The position of the fluorescence maxima of Trp is strongly affected by the polarity of its surrounding, promoting emission from both the ^1^L_a_ and ^1^L_b_ electronic states. In polar solvents, emission of Trp is believed to occur from ^1^L_a_ state, while in non-polar media, the ^1^L_b_ state may have the lower energy, resulting in a blue shift of Trp fluorescence [17]. Meanwhile, according to data from Table 3, in the systems under study, only small shifts of the maxima can be observed with a rather wide variation in polarities.

Thus, the fluorescence quantum yields of (*S,S*)-, (*S,R*)-, and (*R,S*)-optical configurations of KP-Trp dyad in acetonitrile indicates a significant quenching of Trp in the dyad (φ_fl_ NAcTrp = 0.16). As (*R,S*)- and (*S,R*)-enantiomers show identical CIDNP effects reflecting identical ET efficiency, the differences in the fluorescence quantum yields of these enantiomers can be explained by the different efficiency of the singlet–singlet energy transfer (RET) [4,15]. Comparing the CIDNP effects and fluorescence quantum yields of all three considered configurations of KP-Trp dyad one can conclude that the main quenching channel of excited singlet state is RET. 

To establish the contributions of the ET and RET mechanisms to the excited state quenching of D-(*R*)- and L-(*S*)-Trp in the KP-Trp dyad we compared the CIDNP data, containing information on the contribution of the ET and Trp fluorescence quantum yields, reflecting the entire quenching processes. Assuming that the rate constants of electron transfer in (*R,S*)- and (*S,R*)-enantiomers are the same and the main quenching channel of Trp singlet excited state in the dyad is RET, the rate constants of energy transfer can be estimated from the data on the quenching of fluorescence as follows [14]:

(3) $$\frac{\varphi_{0}}{\varphi }=1+k_{q}\tau_{0},$$

where *φ*_0_ and τ_0_ are the fluorescence quantum yield and lifetime of the free Trp (0.16 and 3.6 ns), *φ* is the fluorescence quantum yield of Trp in the dyad, and *k_q_* is the quenching constant (*k_q_* = *k_ET_*+*k_RET_*). Then, the ratio of the rate constants of quenching by the Förster mechanism in the *(R,S)*- and *(S,R)*-enantiomers in acetonitrile is 1.6, while the ratio of quenching constants of (*R,S*)- and (*S,S*)-diastereomers is 2. The summary data with the ratios of ET and RET channels for three optical configurations are presented in Table 4.

As follows from Table 4, (*R,S*)- and (*S,R*)-enantiomers differ in RET, while (*S,S*) and (*R,S*)-diastireomers differ both in ET and RET.

### 2.4. Modern Concepts of the Mutual Influence of Chiral Centers

Thus, it turns out that measurements of quantum yields and kinetics of fluorescence of *(S,R)*- and *(R,S)*-enantiomers have demonstrated differences, contrary to CIDNP data and the concepts existing in stereochemistry. However, examples of different medical activity of drug enantiomers with multiple chiral centers are well known in pharmacology [18,19]. In particular, the authors of [19] provides evidence demonstrating multiple differences in the therapeutic efficacy of isomers of the known beta blocker nebivolol. The drug is a racemic combination of D-nebivolol (+*SRRR* nebivolol) and L-nebivolol (–*RSSS)*. In the case of L- and D-nebivolol, the differences in activity are likely due to the peculiarities of interaction with other chiral particles, namely, amino acid residues in the active sites of the receptors to which nebivolol contacts. 

The difference in the efficiency of the D- and L-Trp excited state quenching in dyad by the RET mechanism suggests that two chiral centers in the enantiomer also influence each other. It should be noted that the mechanism of this influence is not known to date. Nevertheless, even from this study using the simplest model—the dyad with two chiral centers, one can already draw a rather significant conclusion. This is the fact that a change in the configuration in the structure with two chiral centers already leads to a difference in the reactivity of the dyad, in terms of quenching the excited state by the energy transfer mechanism. In addition, the “homo” *(S,S)-* and “hetero” *(R,S)-, (S,R)*-configurations differ by the electron transfer efficiency.

Further, we will consider quantum-chemical calculations of dyads conformations in order to identify possible reasons for differences in the rate constants of both energy transfer in (*R,S*)- and (*S,R*)-enantiomers and electron transfer in (*S,S*)- and (*R,S*)/(*S,R*)-diastereomers (see Supplementary Figures S7–S13). The theory of Förster predicts that

(4) $$k_{RET}=\frac{1}{\tau_{D}}{\left(\frac{R_{0}}{r}\right)}^{6},$$

where *R*_0_^6^ = 8.79 × 10^−5^(κ^2^n^−4^φ_D_J(λ)), φ_D_ and τ_D_—fluorescence quantum yield and lifetime of donor, *κ*^2^—is the factor describing donor–acceptor orientation, n—refractive index of the medium, J(λ)—overlap integral between the donor emission and the acceptor absorption.

Therefore, the difference in ET and RET may be accounted for difference in distance and orientation of KP and Trp in different dyad configuration. Analysis of the dependences of the heat of formation of diastereomers on the six torsion angles around the C-C bonds of the bridge and on distances between C = O group of KP and NH group of indole showed that slightly larger distances are characteristic in (*S,R*)- in comparison with (*R,S*)-, while (*S,S*) and (*R,S*) have comparable distances (Table 5).

As, according to Förster theory, the rate constant of RET depends on 1/r^6^, it allows one to estimate the difference in distances between donor (Trp) and acceptor (KP) in (*S,R*)- and (*R,S*)-enantiomers as ^6^√1.6 = 1.08. Meanwhile, the estimates made on the basis of a comparison of the averaged distances from Table 5 give ratio (*S,R*)/(*R,S*) = 1.02. At the same time, interplane angles between chromophores are sufficiently different for (*S,R*). Today, we have no explanation for this fact. Besides, a large, almost an order of magnitude, difference between the values of the hyperpolarization of the *(S,S)* and *(R,S,)-* configurations of the KP-Trp dyad force us use not only average calculated geometric characteristics of dyad, but also values for individual configurations. Therefore, only using the smallest interplane angle with an average distance in the case of the (*R,S*)-configuration leads to the maximum effect of magnetic dipole–dipole interaction on (*R,S*)- that coincides with experiment (see Section 2.2 and Supplementary Material, Section S6).

## 3. Materials and Methods

### 3.1. Synthesis

The synthesis of KP-Trp dyads (Figure S1) with L- and D-Trp was performed according to a method described earlier [14]. The syntheses of *(S,R)* and *(S,S)* dyads were carried out according to the same technique using S-KP and L- and D-Trp; the synthesis of *(R,S)* dyad was made using KP racemate. An Agilent 1260 Infinity HPLC technique with a diode array detector was used for purification and analysis of purity of individual isomers. A semi-preparative column (Diaspher-110-C18, 10 × 250 mm, 5 μm, BioChemMack ST Ltd., Moscow, Russia) was used for purification of individual isomers. The purification was carried out using a mobile phase composed of acetonitrile (ChimMed, Moscow, Russia) and water (50:50) at a flow rate of 4 mL/min with detection at 260 nm. The injection volumes were 250 μL. The purity of isolated isomers was analyzed on a C18 column (Diaspher-110-C18, 2 × 120 mm, 5 μm, BioChemMack ST Ltd., Moscow, Russia). The analyses were carried out using a mobile phase composed of acetonitrile and water (38:62) at a flow rate of 0.7 mL/min with detection at 260 nm. The injection volume was 5 μL.

The purities of individual isomers isolated from racemate mixture were 99.9 % for *(R,S)* and *(S,S)*, respectively. Each purified isomer contained only the other isomer as an impurity. For details see the Supplementary Materials

### 3.2. Optical Spectroscopy 

All UV spectroscopic measurements were performed using quartz cuvettes of 1 cm optical length. Acetonitrile (Kriochrome, Saint Petersburg) and ethylacetate (Eocos-1, Moscow, Russia) were used as solvents. Spectra and kinetic curves of luminescence were recorded with an Edinburgh Instruments FLSP-920 spectrofluorimeter with either a Xenon lamp or laser diode EPLED-270 (λ_ex_ = 270 nm, pulse duration 0.6 ns) as excitation source.

The kinetic traces were fitted by exponential decay functions using a reconvolution procedure. Processing of kinetic curves (programs of Edinburg Instruments—DATA PROCESSING, and FAST) together with IRF due to mathematical convolution allowing the determination of the times of photophysical processes with a resolution of about 100 ps. The absorption spectra were recorded using an Agilent 8453 spectrophotometer.

### 3.3. NMR Measurements

^1^H NMR and 2D COSY spectra were obtained on an Avance HD III NMR spectrometer (Bruker, Germany, 500 MHz ^1^H operating frequency, P(π/2) = 10 μs). CIDNP experiments were performed on a DPX-200 NMR spectrometer (Bruker, Germany, 200 MHz ^1^H operating frequency, P(π/2) = 2.5 μs). A Lambda Physik EMG 101 MSC eximer laser was used as a light source (308 nm, 100 mJ at output window, 20 mJ/pulse in sample volume, pulse duration 15 ns) in the CIDNP experiments. The samples in standard 5 mm Pyrex NMR tubes were irradiated directly in the NMR probe of DPX-200 NMR spectrometer. The samples were bubbled with argon for 10–15 min to remove dissolved oxygen just before photolysis.

Acetonitrile-d_3_ (Aldrich, D 99.8%, St. Louis, MO, USA) was used as solvent.

### 3.4. Pseudo Steady-State Photo-CIDNP (PSS)

The PSS experiments were performed using a DPX-200 NMR spectrometer and a standard pulse sequence: presaturation–delay 1–pulse τ(π)–delay2 (16 laser flashes with repetition rate 50 Hz during delay2)–observation pulse τ(π/2)–acquisition. Delay1/delay2 ≈ 1.1 to remove residual signals of solvents and solutes. After laser irradiation, the ^1^H spectra of products were recorded on Avance HD III NMR spectrometer.

## 4. Conclusions

It was shown that the dyads in which L- and D-Trp were bound to (*R*)/(*S*)-ketoprofen: *(R,S)-* and *(S,R)-*configurations had completely identical NMR spectra and demonstrated identical efficiency of quenching the Trp singlet excited state by the ET mechanism, as it should be expected for enantiomers. At the same time, these enantiomers significantly differ in the rate of fluorescence quenching by the Förster mechanism. In general, for all studied configurations of KP-Trp dyad the RET mechanism prevail, and the ET contribution is maximum for the (*S,S*)- configuration. In particular, these results mean that the mutual influence of chiral centers, which are most likely responsible for the differences in the therapeutic effect of drug enantiomers with several chiral centers, also manifests itself in the case of two centers. In addition, a new property of hyperpolarization in chiral systems was discovered: the dependence of the CIDNP efficiency on the magnitude of the electron dipole–dipole interaction.

## Figures and Tables

**Table 1:** Observed CIDNP enhancement coefficients (K) of protons and its ratios K_SS_/K_SR, RS_ for KP-Trp dyad in acetonitrile.

	K(CH_2_)	K(NH)
(*S,S*)	1.91	2.08
(*S,R*), (*R,S*)	0.195	0.255
K_SS_/K_SR,RS_	9.8	8.2
Average	9.0	

**Table 2:** Fluorescence lifetimes for three optical configurations of the KP-Trp dyad at different dielectric constants of the solvent (mixtures of acetonitrile/ethylacetate).

	(*R,S*)-KP-Trp				(*S,R*)-KP-Trp				(*S,S*)-KP-Trp			
ε	τ_1_, ns	A_1_, %	τ_2_, ns	A_2_, %	τ_1_, ns	A_1_, %	τ_2_, ns	A_2_, %	τ_1_, ns	A_1_, %	τ_2_, ns	A_2_, %
36.8	0.2 ± 0.1	49	4.5 ± 0.5	51	0.1 ± 0.1	27	4.4 ± 0.4	73	0.1 ± 0.1	50	4.3 ± 0.4	50
30.25	0.2 ± 0.1	55	4.6 ± 0.5	45	0.2 ± 0.1	27	4.3 ± 0.4	73	0.1 ± 0.1	44	4.1 ± 0.4	56
23.85	0.1 ± 0.1	57	4.4 ± 0.5	43	0.2 ± 0.1	27	4.2 ± 0.4	73	0.1 ± 0.1	49	4.2 ± 0.4	51
17.7	0.2 ± 0.1	57	4.4 ± 0.4	43	0.2 ± 0.1	26	4.1 ± 0.4	74	0.1 ± 0.1	51	4.1 ± 0.4	49
11.9	0.2 ± 0.1	56	4.4 ± 0.4	44	0.2 ± 0.1	24	4.0 ± 0.4	76	0.2 ± 0.1	45	4.0 ± 0.4	55
6.19	0.2 ± 0.1	51	4.1 ± 0.4	49	0.3 ± 0.1	26	3.7 ± 0.4	74	0.2 ± 0.1	50	3.9 ± 0.4	50

**Table 3:** Fluorescence quantum yields (φ_fl_) and emission maxima (λ_em_) for three optical configurations of the KP-Trp dyad at different dielectric constants of the solvent (mixtures of acetonitrile/ethylacetate).

	(*R,S*)-KP-Trp		(*S,R*)-KP-Trp		(*S,S*)-KP-Trp	
ε	φ_fl_	λ_em_, nm	φ_fl_	λ_em_, nm	φ_fl_	λ_em_, nm
36.8	0.0027 ± 0.0003	327	0.0043 ± 0.0004	328	0.0052 ± 0.0005	328
30.25	0.0025 ± 0.0003	325	0.0049 ± 0.0005	328	0.0061 ± 0.0006	328
23.85	0.0025 ± 0.0003	325	0.0049 ± 0.0005	328	0.0055 ± 0.0006	328
17.7	0.0026 ± 0.0003	326	0.0049 ± 0.0005	328	0.0052 ± 0.0005	328
11.9	0.0026 ± 0.0003	326	0.0054 ± 0.0005	326	0.0058 ± 0.0006	327
6.19	0.0026 ± 0.0003	325	0.0049 ± 0.0005	326	0.0052 ± 0.0005	325

**Table 4:** The ratios of k_ET_ and k_RET_ in enantiomers and diastereomers of KP-Trp.

	k_ET_	k_RET_
(*R,S*)/(*S,R*)	1	1.6
(*S,S*)/(*R,S*)	(4.7 ± 3.4) *	0.5

* In this case, taking into account the wide variation in the contribution of spin selectivity to the hyperpolarization of the *(S,S)* and *(R,S)* diastereomers of the dyad, we can only make a rough estimate of the contribution ratio of the ET mechanism.

**Table 5:** Average distances and interplane angles in three optical configurations of KP-Trp dyad obtained from Hyperchem models (see Supplementary Figures S7–S13).

	(*R,S*)	(*S,R*)	(*S,S*)
Distance, Å	4.11	4.19	4.13
Interplane angle, °	32.3	56.3	30.1

## Data Availability

Not applicable.

## Supplementary Materials

The following are available online at https://www.mdpi.com/article/10.3390/ijms22126198/s1, Figure S1: structures of KP-Trp dyads; Section 2: purification of chiral dyads; Figures S2–S4: 2D COSY of (*S,S*)-, (*S,R*)- and (*R,S*)-KP-Trp in acetonitrile-d3, Figures S5–S6: ^1^H NMR spectra of 5 mM (*S,S*)- and (*S,R*)-KP-Trp in acetonitrile-d3 before and after photolysis, Table S1: CIDNP coefficients (K = I_pol_/I_eq_) of (*R,S*)- and (*S,S*)-diastereomers of NPX-Trp dyad in acetonitrile/benzene mixture (ε = 14.5), Figure S7: Structure of KP-Trp dyad with marked up rotating bonds (1–6) and carbonyl carbon of KP and nitrogen atom of indole fragment, Figures S8–S13: The dependences of: heat of formation, interplane angle and intercenter distance on the values of torsion angle (1) (see Figure S7) calculated for (**a**) (*R,S*)-,(**b**) (*S,S*)- and (**c**) (*S,R*)-KP-Trp.
