# Supplementary material for: Role of Chiral Configuration in the Photoinduced Interaction of D- and L-Tryptophan with Optical Isomers of Ketoprofen in Linked Systems
Source: Int J Mol Sci. 2021 Jun 8;22(12):6198. doi: 10.3390/ijms22126198 (PMC8227724; doi:10.3390/ijms22126198)
Supplement: Supplementary file 1 [file ijms-22-06198-s001.zip › ijms-1246617-supplementary.pdf]

Supplementary Materials

1. Structures of the systems under study

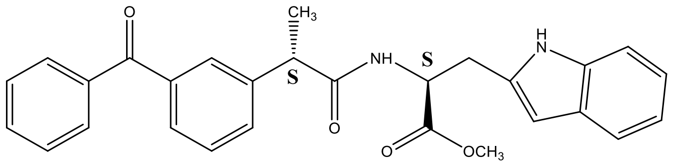

S,S-KP-Trp

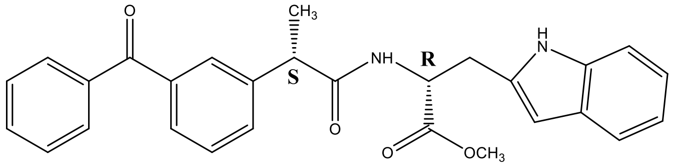

S,R-KP-Trp

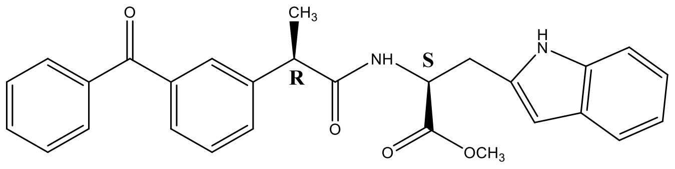

R,S-KP-Trp

Figure S1. Chiral centers are designated according to the Cahn - Ingold - Prelog system.

2. Purification of KP-Trp dyad

Purification of the compounds was carried out by chromatography on Al_2O_3 + 5% Zn_2SIO_4-Mn, eluent EtOAc/hexane - 1:1. The sorbent zone with Rf = 0.8 was collected. The product was eluted from the sorbent by EtOAc and the solvent was removed under reduced pressure (50 °C, 15 mm Hg). The obtained oily liquid was re-chromatographed under the same conditions. After removing the solvent under reduced pressure (50 °C, 15 mm Hg), the resulting yellow oil was evacuated at room temperature at 1 mm Hg for 7 h to get white amorphous powder, m.p. 52–54°C. Further purification was carried out using a mobile phase composed of acetonitrile (ChimMed, Moscow, Russia) and water (50:50) at a flow rate of 4 mL/min with detection at 260 nm. The injection volumes were 250 µL. The purity of isolated isomers was analyzed on a C18 column (Diaspher-110-C18, 2 x120 mm, 5 µm, BioChemMack ST Ltd, Moscow, Russia). The analyses were carried out using a mobile phase composed of acetonitrile and water (38:62) at a flow rate of 0.7 mL/min with detection at 260 nm. The injection volume was 5 µL.

The purities of individual isomers isolated from racemate mixture were 99.9 %. ^{13}C NMR of (S,S) optical isomer (T = 300 K, CD_3CN) δ 17.92, 27.06, 45.63, 51.64, 53.20, 109.68, 111.39, 118.27, 119.03, 121.59, 123.62, 127.46, 128.39, 128.41, 128.56, 128.94, 129.82, 131.64, 132.52, 136.39, 137.53, 137.67, 142.11, 172.14, 173.15, 196.17.

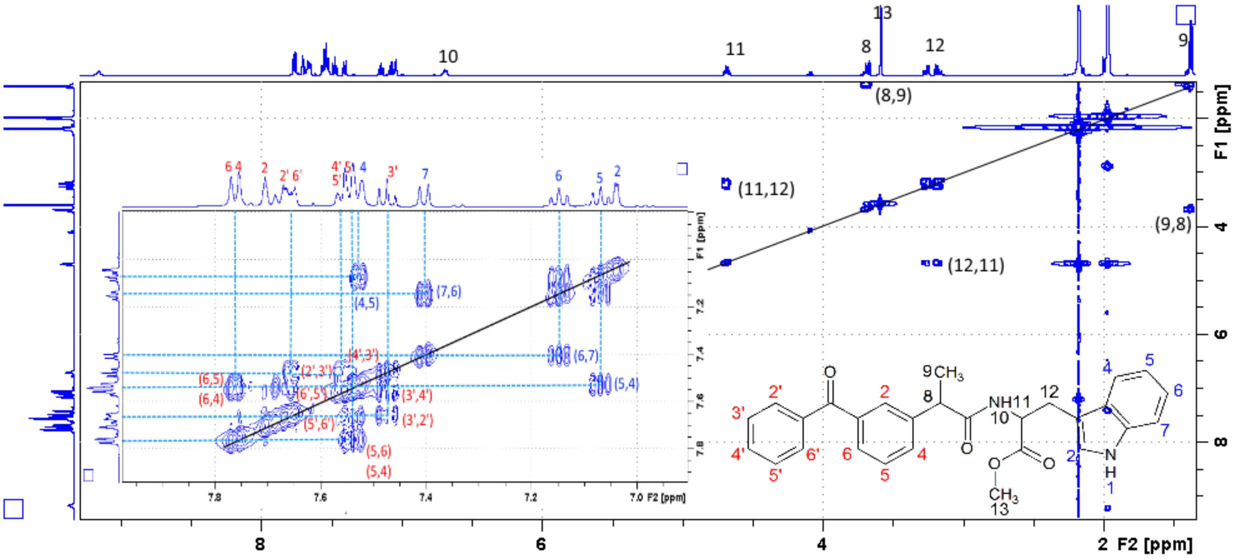

Figure S2. 2D COSY of (*S,S*)-KP-Trp in acetonitrile-d3.

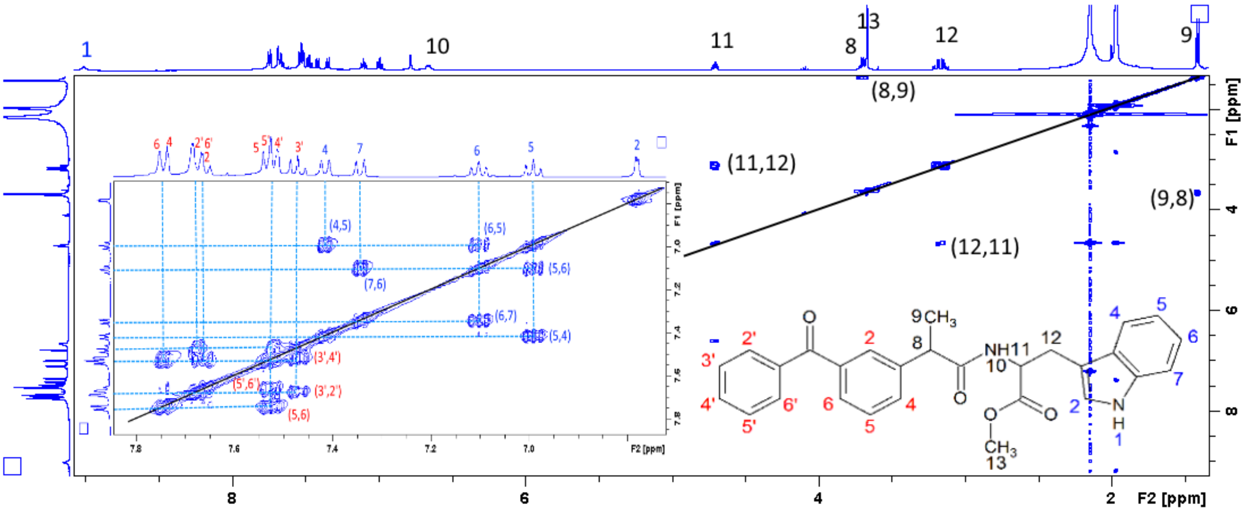

Figure S3. 2D COSY of (S,R)-KP-Trp in acetonitrile-d3.

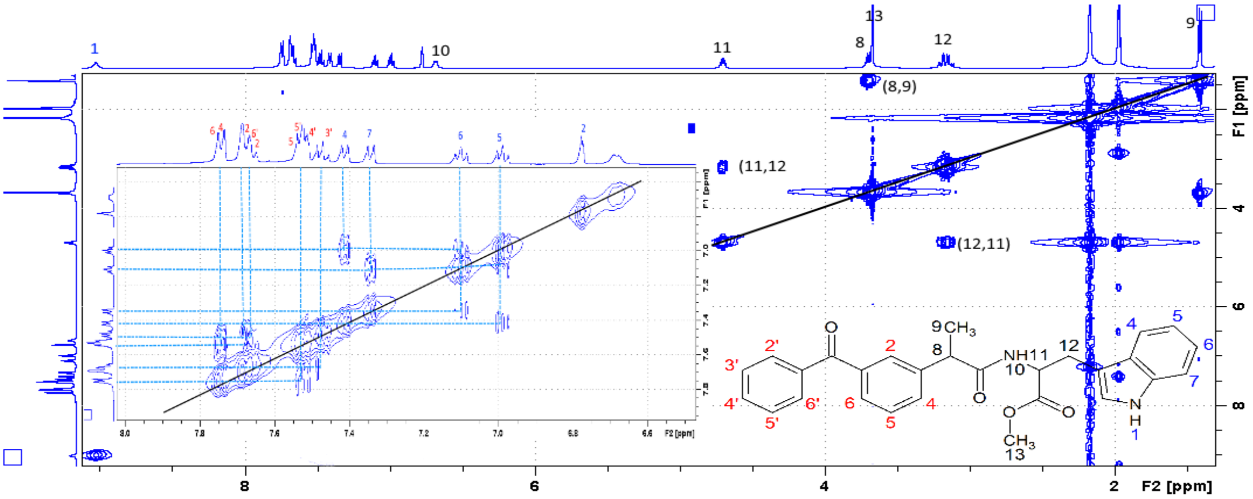

Figure S4. 2D COSY of (R,S)-KP-Trp in acetonitrile-d3.

3. NMR spectra of photolysis products of KP-Trp dyad

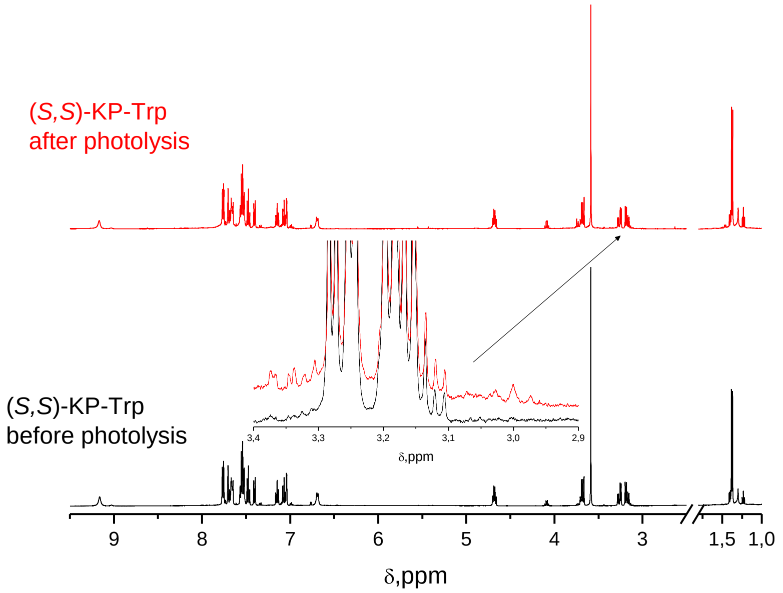

Figure S5. ^{1}H NMR spectra of 5 mM (*S,S*)-KP-Trp in acetonitrile-d3 before and after photolysis. In insert expanded region of CH2 protons.

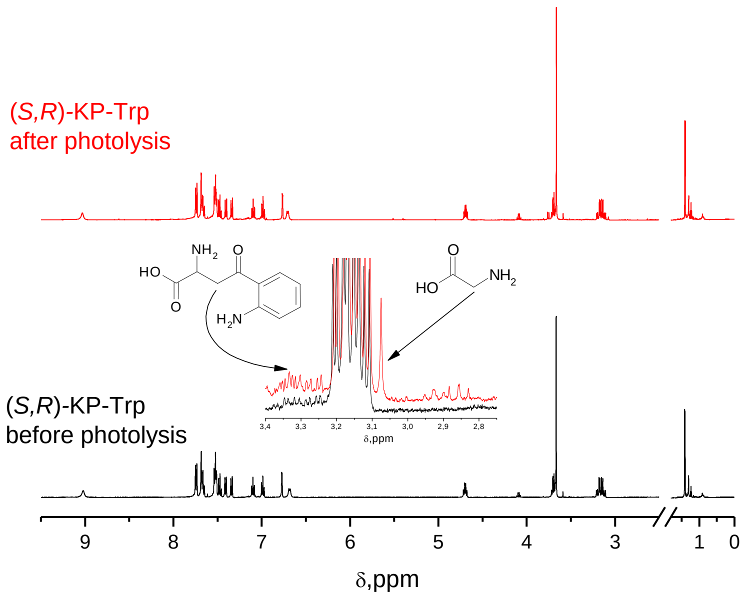

Figure S6. ^{1}H NMR spectra of 5 mM (*S,R*)-KP-Trp in acetonitrile-d3 before and after photolysis. In insert expanded region of CH2 protons with assignment of possible by-products (derivatives of tryptophan): glycine and kynurenine [C. Schöneich, J. Pharm. Pharmacol., 2018, 70, 655].

4. CIDNP coefficients in diastereomers of NPX-Trp dyad

Table S1. CIDNP coefficients ($K = I_{pol}/I_{eq}$) of (*R*,*S*)- and (*S*,*S*)-diastereomers of NPX-Trp dyad in acetonitrile/benzene mixture (ε = 14.5).

	K (CH_2)	K (NH)
HFI, Gs	7.0–10.0	−3.0–3.7
(R,S)	3.67	12.12
(S,S)	2.21	6.68
**K_{RS}/K_{SS}**	**1.7**	**1.8**
**Average**	**1.75**	

5. Quantum chemical calculations of possible conformations of (S,S)-,(R,S)- and (S,R)-optical isomers of KP-Trp dyad

Calculations were performed using software Hyperchem 8 by semi-empirical AM1 method. Energy of dyads spatial configuration was minimized with restriction of selected torsion angle and optimization of all other geometrical parameters. The figures presented below show the calculations results of the dependence of the values of the formation heat, the distances between donor and acceptor of dyad's diastereomers, and the angle between the planes of the ketoprofen and tryptophan fragments upon rotation of six bonds (shown in Figure S7). This is specifically the distances between carbonyl carbon of KP and nitrogen atom of indole fragment of dyad (inter-center distance) and the angles between planes of Trp aromatic ring and "C-COC" fragment of KP (interplane angle) calculated for different torsion angles.

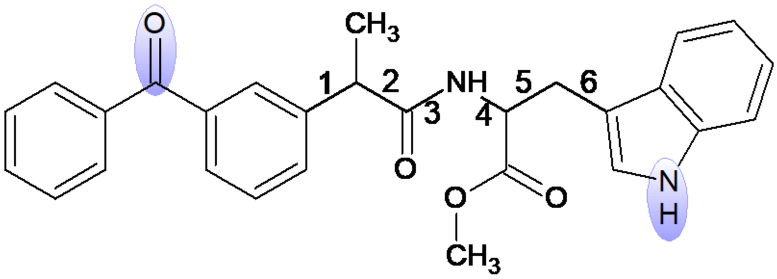

Figure S7. Structure of KP-Trp dyad with marked up rotating bonds (1–6) and carbonyl carbon of KP and nitrogen atom of indole fragment.

In the Figures S8–S13 below blue rectangles represent areas with the lowest heat of formation, i.e. more stable conformations and corresponding interplane angles and distances, the red line is the average value of interplane angles and distances.

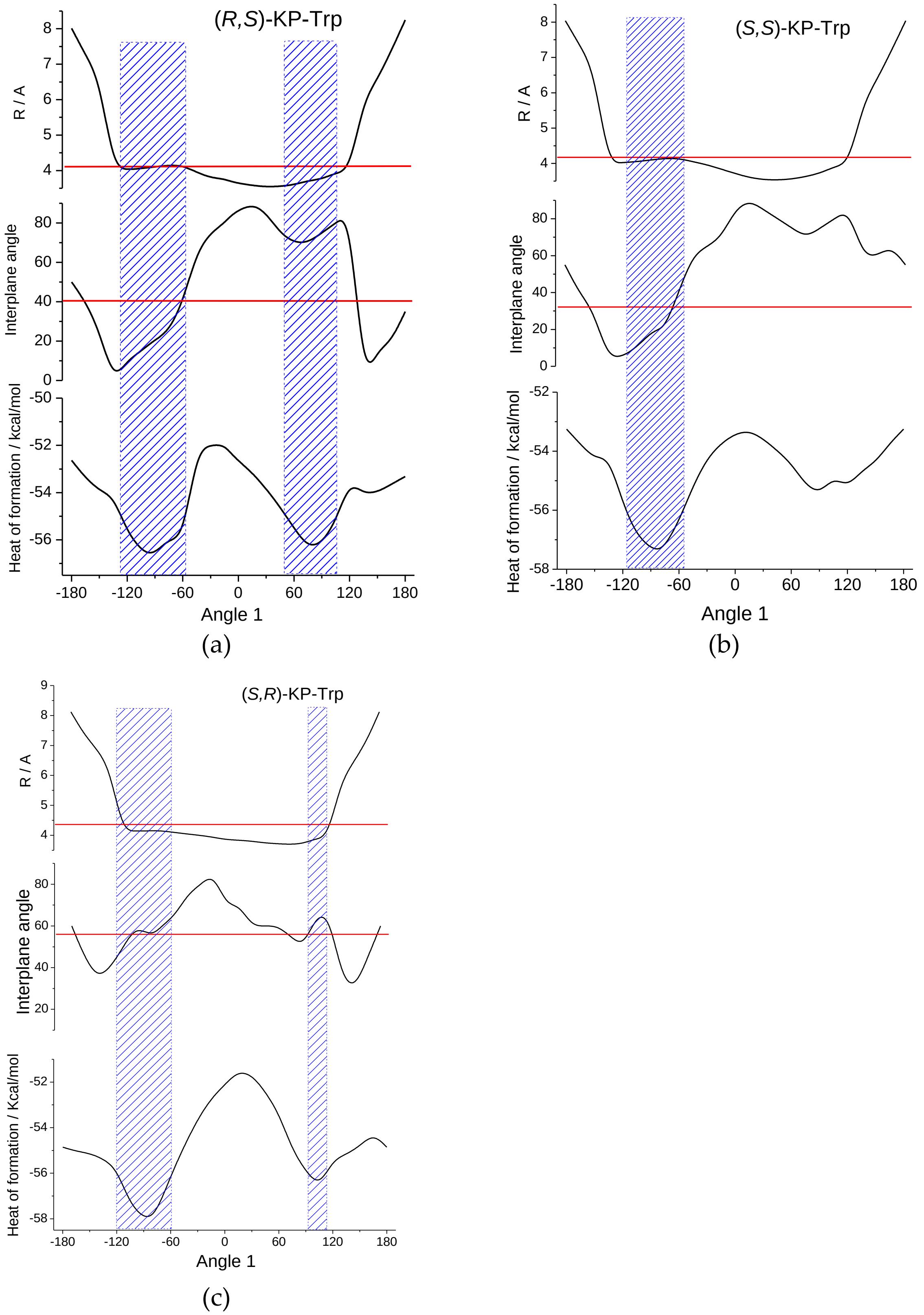

Figure S8. The dependences of: heat of formation, interplane angle and intercenter distance on the values of torsion angle (1) (see Figure S7) calculated for **(a)** (*R*,*S*)-,**(b)** (*S*,*S*)- and **(c)** (*S*,*R*)-KP-Trp.

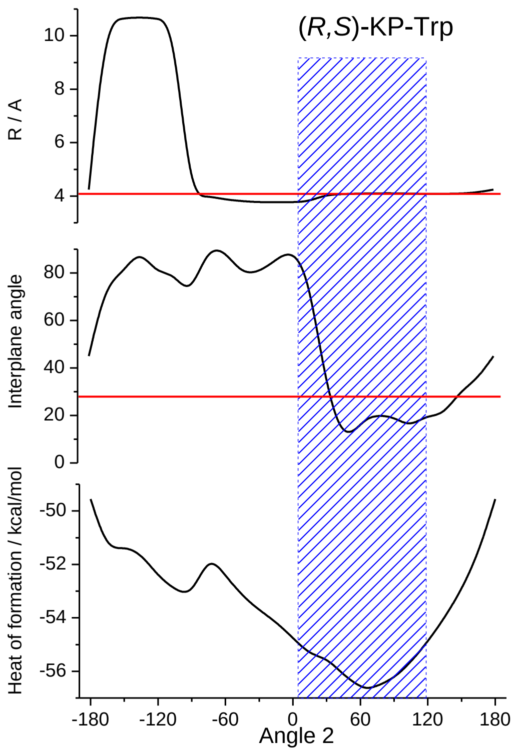

(a)

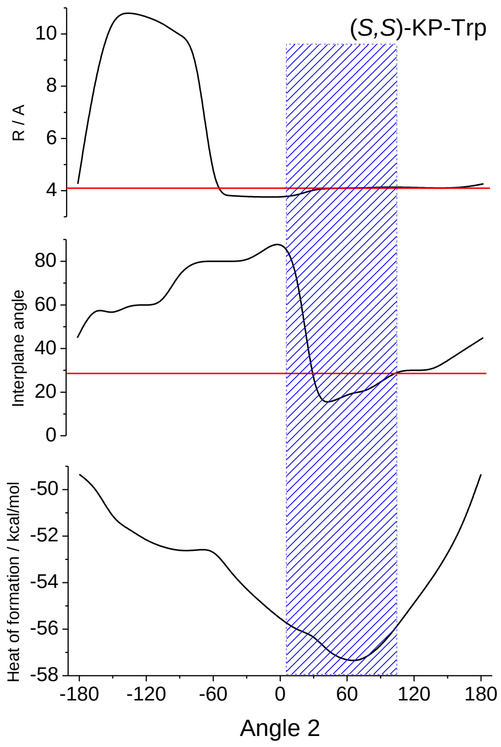

(b)

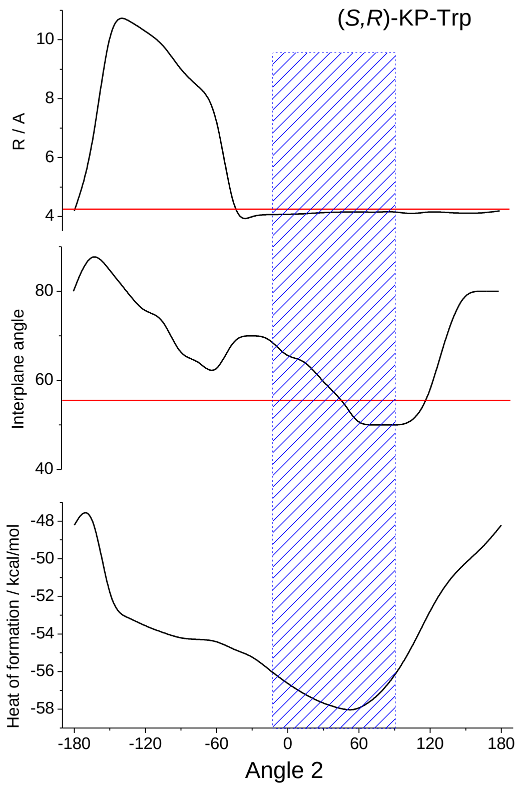

(c)

Figure S9. The dependences of: heat of formation, interplane angle and intercenter distance on the values of torsion angle (2) (see Figure S7) calculated for **(a)** (*R*,*S*)-,**(b)** (*S*,*S*)- and **(c)** (*S*,*R*)-KP-Trp.

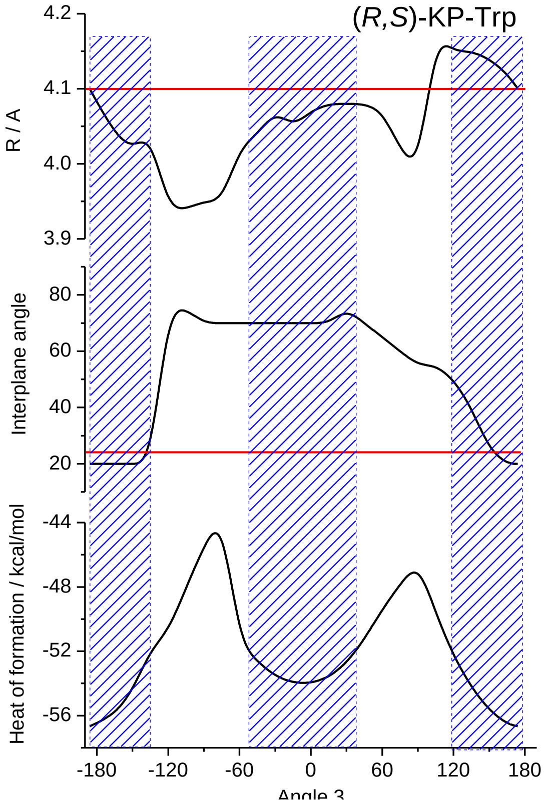

(a)

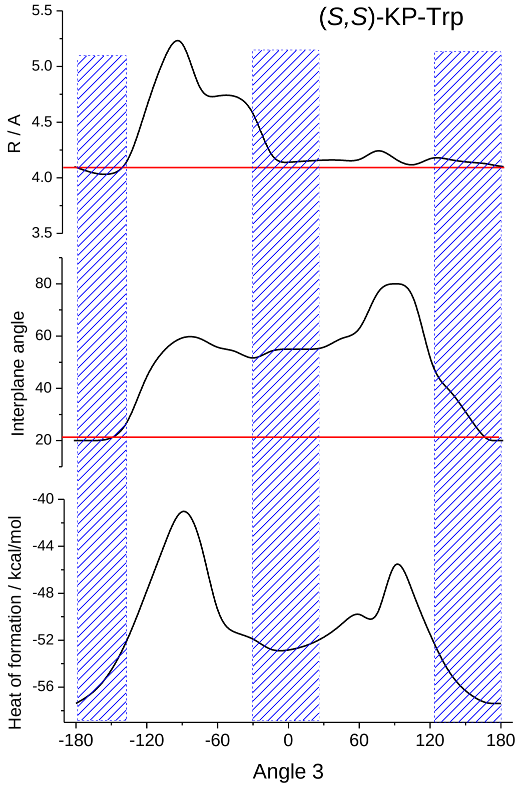

(b)

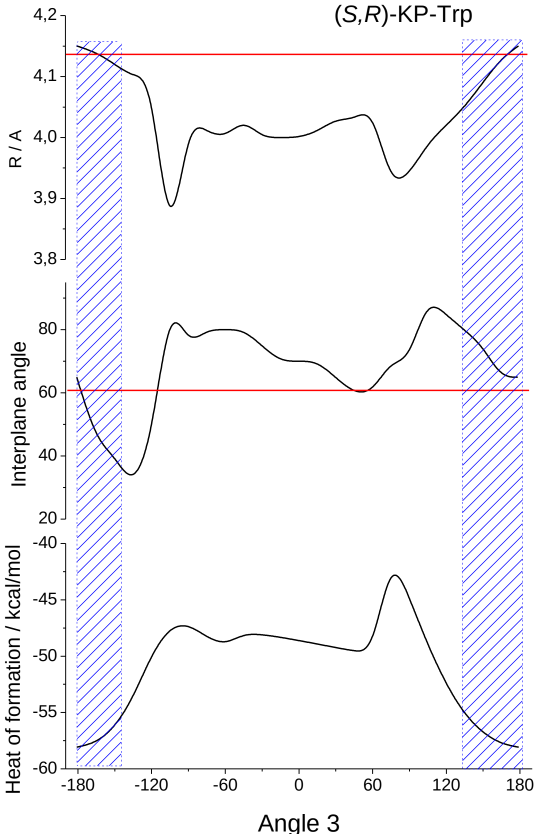

(c)

Figure S10. The dependences of: heat of formation, interplane angle and intercenter distance on the values of torsion angle (3) (see Figure S7) calculated for **(a)** (*R*,*S*)-,**(b)** (*S*,*S*)- and **(c)** (*S*,*R*)-KP-Trp.

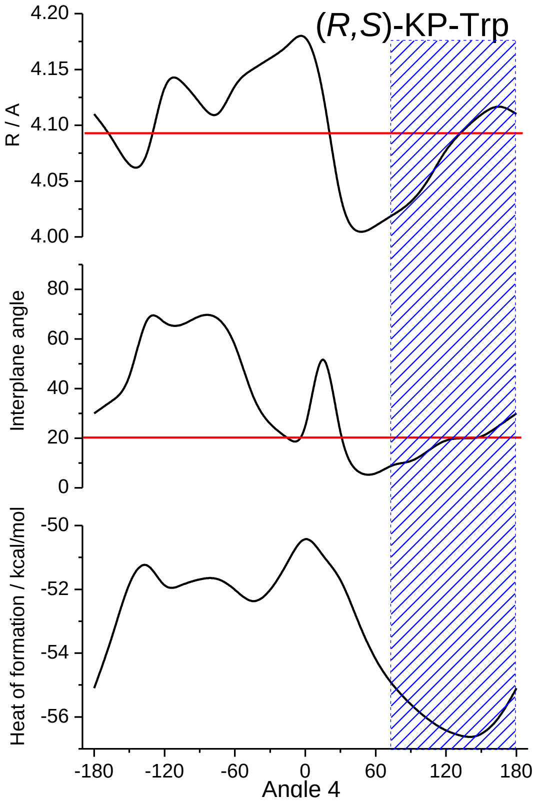

(a)

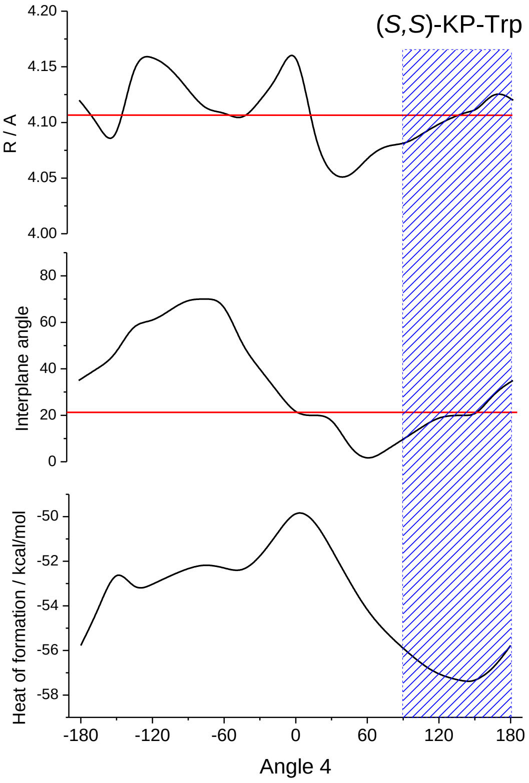

(b)

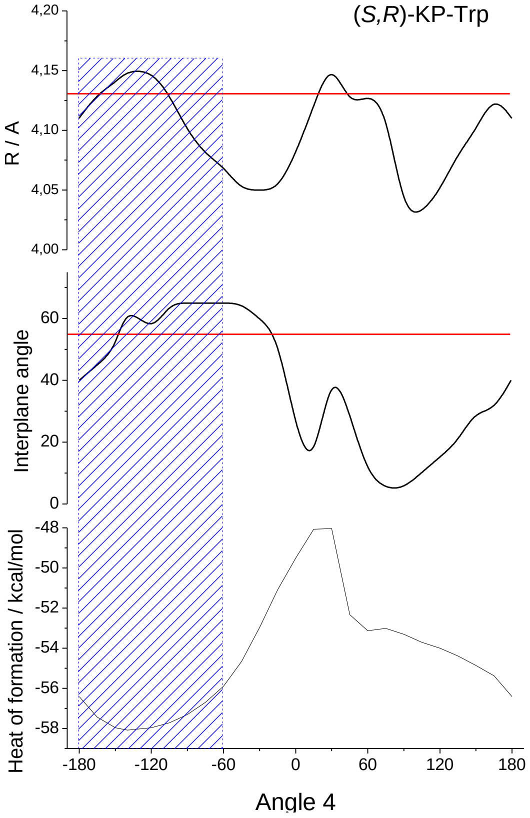

(c)

Figure S11. The dependences of: heat of formation, interplane angle and intercenter distance on the values of torsion angle (4) (see Figure S7) calculated for **(a)** (*R*,*S*)-,**(b)** (*S*,*S*)- and **(c)** (*S*,*R*)-KP-Trp.

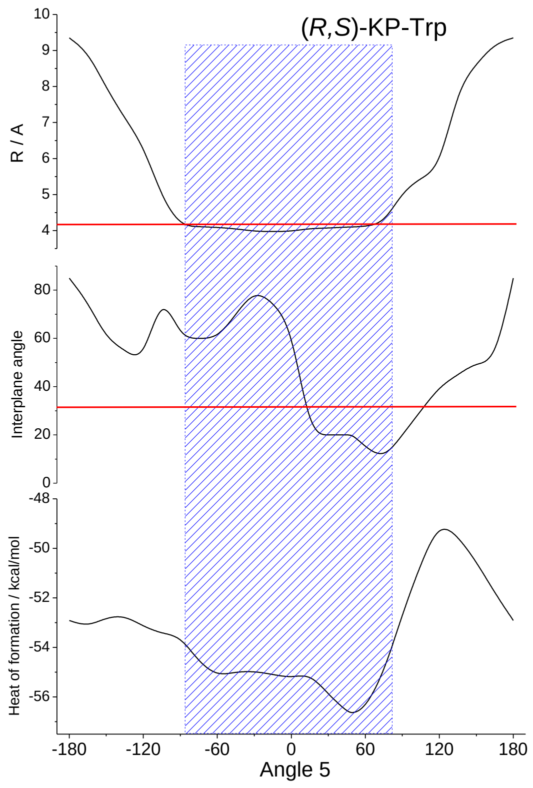

(a)

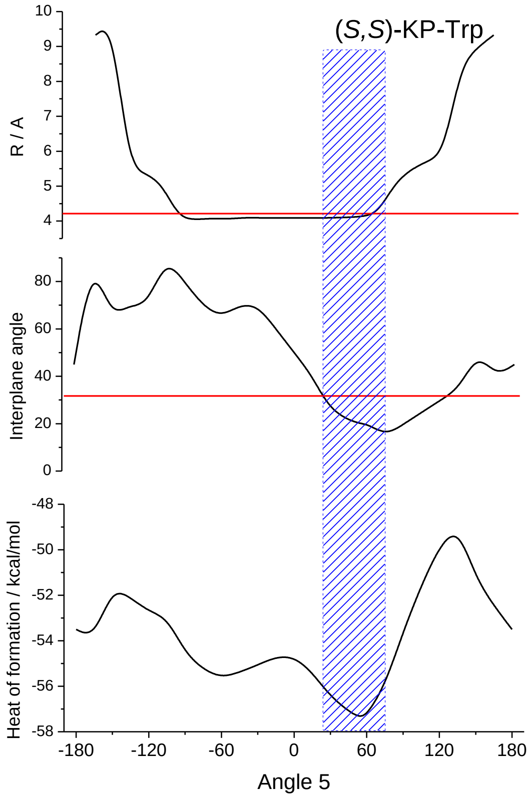

(b)

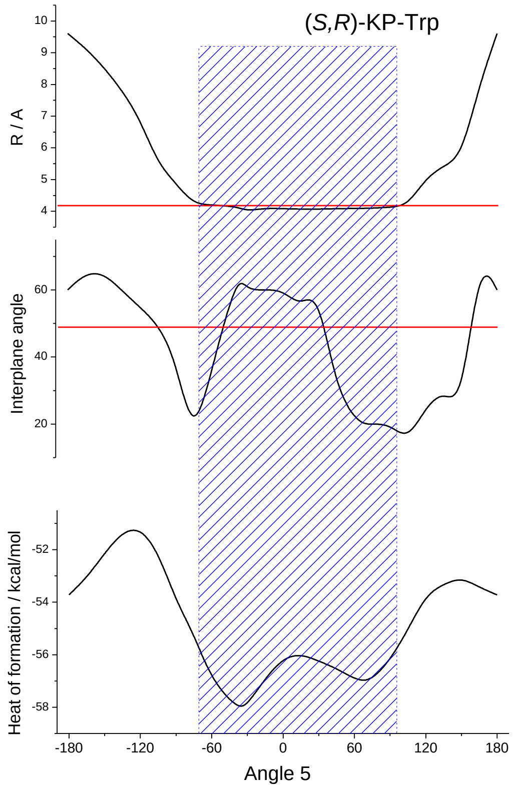

(c)

Figure S12. The dependences of: heat of formation, interplane angle and intercenter distance on the values of torsion angle (5) (see Figure S7) calculated for **(a)** (*R*,*S*)-,**(b)** (*S*,*S*)- and **(c)** (*S*,*R*)-KP-Trp.

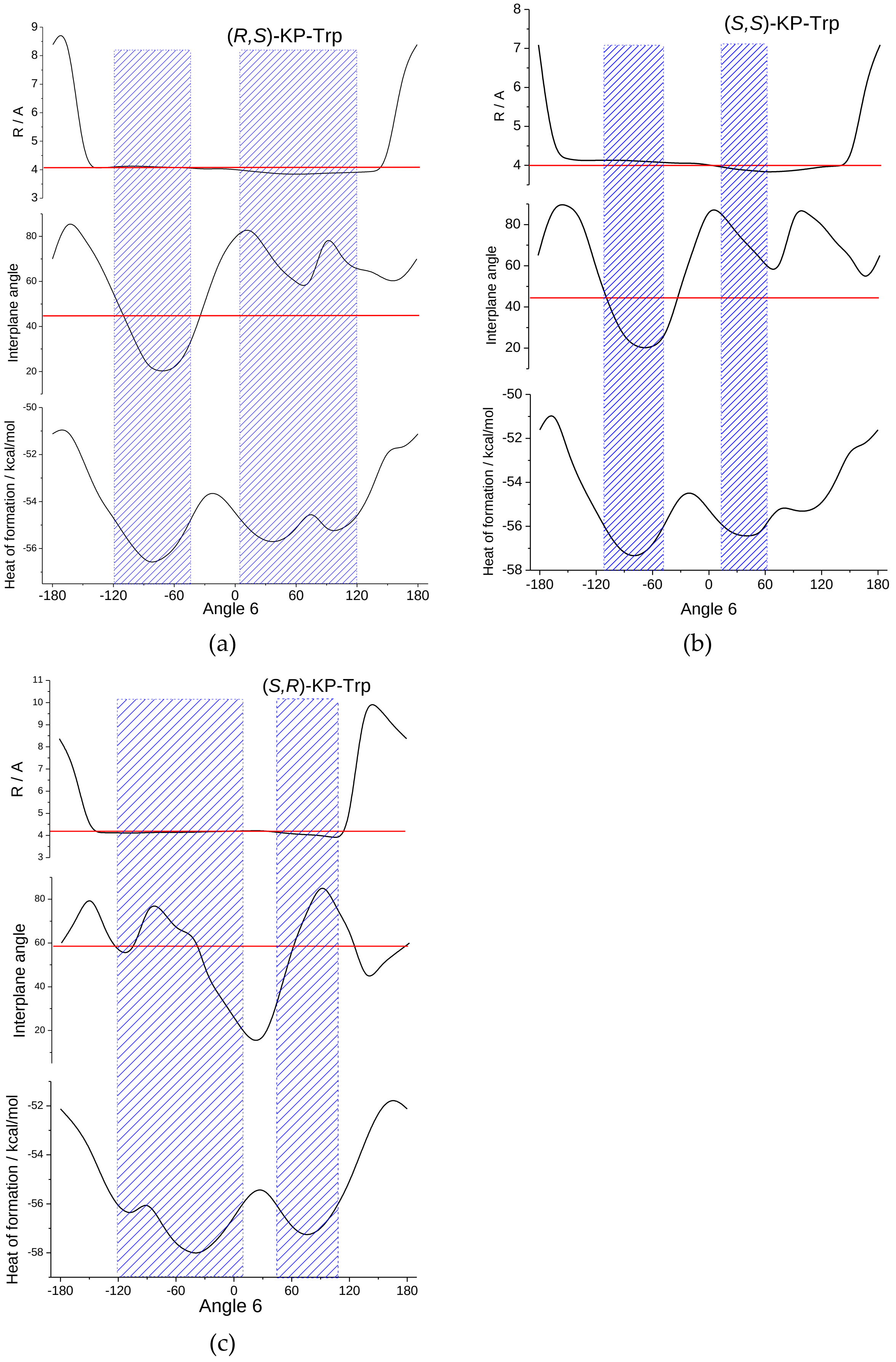

Figure S13. The dependences of: heat of formation, interplane angle and intercenter distance on Table 6. (see Figure S7) calculated for **(a)** (*R*,*S*)-,**(b)** (*S*,*S*)- and **(c)** (*S*,*R*)-KP-Trp.

6. Magnetic dipole-dipole interaction of electrons (theory and calculations)

To elucidate the influence of the magnetic dipole-dipole interaction on the value of the stationary nuclear polarization, solutions of the equations for the elements of the stationary density matrix are found that describe quantum transitions between the singlet S and triplet T_0 spin states under the action of spin interactions. This is the interaction (Zeeman) of the dyad's electrons with an external strong magnetic field, HFI and magnetic dipole-dipole interaction of electrons. The Zeeman interaction of nuclei with an external magnetic field and mobility of reactants were neglected.

Let us introduce dimensionless parameters

$$Z = \frac{\left(k_S + k_T\right)^2 \left(\delta^2 + a^2\right)}{k_S k_T \left(\left(\frac{k_S + k_T}{2}\right)^2 + A^2\right)}, \qquad \Delta = \frac{2\delta a}{\delta^2 + a^2}, \quad \xi = \frac{1}{2T\left(k_S + k_T\right)}, \tag{1}$$

where

δ - the difference in the Larmor frequencies of the biradical electrons,

a - hyperfine interaction constants of electrons of the biradical with their nuclei (it is assumed that each electron interacts with one nucleus with equal HFI constants),

$k_S = k_{BET}$ - the rate of transformation of the biradical in the singlet state,

$k_T = k_{T'} + k_p + \frac{1}{T_1} \equiv k_{T'} + \frac{1}{T}$ $\left(\frac{1}{T} = k_p + \frac{1}{T_1}\right)$ - the rate of transformation of the biradical in the triplet state,

$k_{T'}$ - the transformation rate of the triplet state that contributes to the required polarization,

k_p - the rate of product formation that does not contribute to the required polarization,

T_1 - relaxation time of triplet state,

$\frac{1}{T}$ - the total transformation rate of the triplet state that does not contribute to the required polarization,

$A = \frac{D}{r^3}\frac{1-3\cos 2\theta}{2\hbar} \equiv \frac{D}{2\hbar}\cdot 10^{22} f$, - matrix element of magnetic dipole-dipole interaction (in units of frequency), where $D = g_1 g_2 \beta^2$, $\beta = 0{,}927{,}4009 \cdot 10^{-20}\, {}^{erg}/_{Gs}$, $\hbar = 1{,}054571 \cdot 10^{-27} erg \cdot s$) (2)

Factor f is defined as

$$f = 100 \cdot \frac{1 - 3\cos^2\theta}{r_A^3}, \tag{3}$$

where r_A - the effective distance between the dyad's electrons in Å, and θ a certain effective polar orientation angle of the radius vector connecting the electrons of the dyad relative to the quantization axis.

Then for the stationary polarization of the first nucleus P_1 and the second nucleus P_2, we have

$$P_1 = -P_2 = -\frac{\xi Z \Delta}{\left(1+Z\right)^2 - Z^2\Delta^2}, \tag{4}$$

(Note that if we mean polarization as the energy of the Zeeman interaction, then the result should be multiplied by $-\gamma_N \hbar B = -\hbar\omega_N$, $(\gamma = \frac{g_N \mu_N}{\hbar})$, where the subscript N denotes the values related to given nucleus.)

The dipole-dipole magnetic interaction leads to the splitting of the spin levels S and T_0, resulting in the decrease of intensity of the singlet-triplet transitions caused by the difference between the Larmor frequencies and HFI. Stationary polarization decrease with increasing the dipole-dipole interaction. Further, in the calculations, we will use the following values of the magneto-resonance parameters:

$$a = 0.7 - 1.0 \text{ m}T = 7 - 10\ G = (5,6-8)\cdot 10^7 s^{-1}$$

$$\delta = \frac{\Delta g \beta}{\hbar} B = 1,6533\cdot 10^7 s^{-1}$$

(g-factors of KP and Tr are 2,0030 and 2,0026, magnetic field $4,7\cdot 10^3$ G)

$$k_S = 2\cdot 10^9 s^{-1}, \quad k_T = 1,8\cdot 10^9 s^{-1}, \quad A = 1,6311\cdot 10^9 s^{-1} f \qquad (5)$$

The parameter ξ for the calculation of the polarization ratio is insignificant if it is the same for both types of dyads. We have, taking for the HFI constant the value $7\cdot 10^7 s^{-1}$

$$\Delta = \frac{2\delta a}{\delta^2 + a^2} \approx \frac{14\cdot 1,6533\cdot}{(1,6533)^2 + 49} = \frac{23,1462}{2,7334+49} = \frac{23,1462}{51,7334} = 0,4474, \quad \Delta^2 = 0,2002 \qquad (6)$$

This parameter is the same in both cases. Parameter

$$Z = \frac{(k_S + k_T)^2 (\delta^2 + a^2)}{k_S k_T \left(\left(\frac{k_S + k_T}{2} \right)^2 + A^2 \right)} \approx \frac{14.44\cdot 51,7334\cdot 10^{32}}{3,6\cdot 10^{18}\left(3,61\cdot 10^{18} + 2,66\cdot 10^{18} f^2\right)} =$$

$$= \frac{14.44\cdot 51,7334\cdot 10^{-4}}{3,6\cdot\left(3,61\cdot + 2,66\cdot f^2\right)} = \frac{0,02075}{3,61\cdot + 2,66\cdot f^2}, \qquad (7)$$

i.e. to calculate the parameter Z, it is necessary to calculate the parameter f.

Calculation of polarizations for the parameters (θ, r) obtained from Hyperchem model.

For (S,S) $\theta = 30,1^\circ$, $r \approx 4,13\, A = 4,13\cdot 10^{-8} cm$, for (R,S) $\theta = 32,3^\circ$, $r_A \approx 4,11\, A = 4,11\cdot 10^{-8} cm$,

Then

$$\cos^2\theta_{RS} = \cos^2 32,3^\circ = 0,7145, \qquad 1 - 3\cos^2\theta_{RS} = -1,1434$$

$$\cos^2\theta_{SS} = \cos^2 30,1 = 0,7485, \qquad 1 - 3\cos^2\theta_{SS} = -1,2455$$

$$r_{ARS}^3 = (4,11)^3 = 69,43, \qquad r_{ASS}^3 = (4,13)^3 = 70,44$$

(8)

$$f_{RS} = -\frac{100\cdot 1,1434}{69,43} = -\frac{114,34}{69,43} = -1,6469, \quad f_{RS}^2 = 2,7123$$

$$f_{SS} = -\frac{100\cdot 1,2455}{70,44} = -\frac{124,55}{70,44} = -1,7680, \quad f_{SS}^2 = 3,1258 \qquad (9)$$

For Z we have

$$Z_{RS} = \frac{0,02075}{3,61\cdot + 2,66\cdot f_{RS}^2} = \frac{0,02075}{3,61\cdot + 2,66\cdot 2,7123} = 0,001917$$

$$Z_{SS} = \frac{0,0275}{3,61\cdot + 2,66\cdot f_{SS}^2} = \frac{0,02075}{3,61\cdot + 2,66\cdot 3,1258} = 0,001740 \qquad (10)$$

The polarization is proportional to the following value

$$I_{RS} = \frac{Z_{RS}}{(1 + Z_{RS})^2 - Z_{RS}^2 \Delta^2} = \frac{0,001917}{(1 + 0,001917)^2 - 0,001917 \cdot 0,2002} \approx 0.001910$$

$$I_{SS} = \frac{Z_{SS}}{(1 + Z_{SS})^2 - Z_{SS}^2 \Delta^2} = \frac{0,001740}{(1 + 0,001740)^2 - 0,001740 \cdot 0,2002} \approx 0.001734$$

$$\frac{P_{SS}}{P_{RS}} = \frac{0.001734}{0.001910} = \frac{173,4}{191,0} \approx 0,908$$

These values are obtained using averaged geometric parameters. Averaging was carried out over all six angles of rotation. If we assume that not all configurations are equally probable, and take separate values of the angles for *S,S* and *R,S,* then we can get a sevenfold difference. $\frac{P_{SS}}{P_{RS}} \approx 7$ at the following geometrical parameters: r_A= 4.1 Å, interplane angles θ_{RS} = 0–5° and θ_{SS}= 53–57°.

Thus, the magnetic dipole-dipole interaction really influence the net polarization .
